# Division and spreading of attention across color

**DOI:** 10.1093/cercor/bhae240

**Published:** 2024-06-10

**Authors:** Jasna Martinovic, Antoniya Boyanova, Søren K Andersen

**Affiliations:** School of Philosophy, Psychology and Language Sciences, University of Edinburgh, 7 George Square, EH8 9JZ, Edinburgh, United Kingdom; School of Psychology, University of Aberdeen, William Guild Building, AB24 3UB, Aberdeen, United Kingdom; School of Psychology, University of Aberdeen, William Guild Building, AB24 3UB, Aberdeen, United Kingdom; Department of Psychology, University of Southern Denmark, Campusvej 55, 5230 Odense, Denmark

**Keywords:** attentional selection, color, divided attention, target-distractor proximity, steady-state visual evoked potentials

## Abstract

Biological systems must allocate limited perceptual resources to relevant elements in their environment. This often requires simultaneous selection of multiple elements from the same feature dimension (e.g. color). To establish the determinants of divided attentional selection of color, we conducted an experiment that used multicolored displays with four overlapping random dot kinematograms that differed only in hue. We manipulated (i) requirement to focus attention to a single color or divide it between two colors; (ii) distances of distractor hues from target hues in a perceptual color space. We conducted a behavioral and an electroencephalographic experiment, in which each color was tagged by a specific flicker frequency and driving its own steady-state visual evoked potential. Behavioral and neural indices of attention showed several major consistencies. Concurrent selection halved the neural signature of target enhancement observed for single targets, consistent with an approximately equal division of limited resources between two hue-selective foci. Distractors interfered with behavioral performance in a context-dependent fashion but their effects were asymmetric, indicating that perceptual distance did not adequately capture attentional distance. These asymmetries point towards an important role of higher-level mechanisms such as categorization and grouping-by-color in determining the efficiency of attentional allocation in complex, multicolored scenes.

## Introduction

Biological systems need to allocate limited processing resources to relevant elements in their environment. Furthermore, many situations require dividing attention amongst multiple feature values: foraging for berries that could range in color between pink, red and dark purple, or monitoring the actions of multiple players in differently colored jerseys as well as the movement of the ball in a fast-paced team sport. In such cases, we can accept that the effectiveness of attentional selection will depend upon the relative positions of attended features within their feature space. For example, if opposing teams wore similar colors, this would make the game difficult to follow.

In vision, selection is achieved through spatial (Posner et al. 1980; Eriksen and St. James 1986), feature-based (Treue and Martinez-Trujillo 1999) or object-based attention (Duncan 1984). It is widely accepted that spatial attention operates by enhancing processing in a confined region of space through application of an attentional focus that has been likened to a spotlight, zoom lens or a Gaussian gradient (Carrasco 2014). Yet, despite decades of research, we still lack a comprehensive model that fully characterizes selection of specific feature values belonging to a continuous domain of a complex, multidimensional feature-space such as color (although see Stormer and Alvarez 2014).

Selection of multiple feature values from the same dimension seems highly consistent between color (Andersen et al. 2013) and space (Toffanin et al. 2009; Adamian et al. 2019; Adamian and Andersen 2022), with reduced attentional modulation in divided attention conditions as proposed by the original Zoomlens model (Eriksen and St. James 1986) and processing of distractors modulated by target-distractor proximity in the feature-dimension on which selection is based (Treue and Martinez-Trujillo 1999; Martinez-Trujillo and Treue 2004; Martinovic et al. 2018). According to Liu and Jigo (2017), divided attention to color is implemented by attending only a single color at a time, which may be due to an inability to maintain multiple active attentional templates for color. This leads to a very clear prediction that attentional modulation in the visual cortices should be halved for each individual target color if attentional switching between the two target templates occurs in a way that ensures that both are afforded equal resources. Such a pattern has recently been reported for division of spatial attention in a multiple-object tracing task (Adamian and Andersen 2022).

Here, we ask the following main question: how does feature-based attention operate when it has to select single or dual targets drawn from the opposite sides of the color space? This question emerges from our recent neuroscientific work on concurrent selection of multiple hues (Martinovic et al. 2018), which demonstrated that different spatially overlaid colors can be attended concurrently with an efficiency that is determined by their configuration in color space. The magnitude of attentional modulation depended overwhelmingly on target proximity and was well described by a simple model which suggested that color space is rescaled in an adaptive manner to support attentional selection. Such adaptive rescaling is consistent with a series of recent studies that show attention optimally biases neural responses depending on several factors: the tuning of sensory neurons, physical characteristics of the visual environment and the nature of the task (Scolari and Serences 2009; Scolari et al. 2012; Verghese et al. 2012). It is also consistent with the recent proposals integrating various evidence streams from the visual search literature (Geng and Witkowski 2019; Yu et al. 2023): attentional guidance only has to be “good-enough” to efficiently direct the observer towards the best approximation of the target in the current sensory context, which is why it is highly dependent on the configuration of targets and distractors in feature space.

Whilst Martinovic et al. (2018) examined the behavioral and neural signature of attentional selection for different sets of dual targets in two different color configurations, the key condition of attending single color targets in the same contexts was missing. Evaluating differences between single target and two-target attentional settings is essential in an attempt to understand the effectiveness of attending to multiple targets (e.g. Liu and Jigo 2017, for a review see Ort and Olivers 2020). Therefore, to address this gap in the literature, we examined the behavioral and neural outcomes of applying single or dual attentional foci in a task that requires sustained feature-based selection. To fully assess how feature-based attentional selection works for individual colors in a display with multiple targets and distractors, it is optimal to be able to record an individual response for each color. We achieved this by using a multicolor random dot kinematogram (RDK) display in combination with frequency-tagging, allowing us to look at the neural markers of attentional processing for four colors simultaneously. This is a well-established procedure for studying the neural markers of feature-based attention (e.g. Andersen and Muller 2010; Andersen et al. 2011a; Andersen et al. 2012; Andersen et al. 2013; Andersen et al. 2015; Gundlach et al. 2023a; Gundlach et al. 2023b). The colors differed in hue but were of constant lightness and colorfulness, as specified in a perceptually uniform color space (CIE Lab; see Fairchild 2013).

We measured neural activity elicited by each color in a 4-color display when attention was focused on red or green or divided between red and green in order to detect infrequent coherent motion events in these colors. To generate different stimulus contexts in which these targets would need to be selected, we manipulated distances between targets and one of the distractor hues. This resulted in three contexts: (i) a putatively neutral context, with intermediate distractors falling between the two target hues (blue and yellow); (ii) a context with one distractor closer to red (orange and yellow); and (iii) a context with one distractor closer to green (lime and yellow). We measured neural and behavioral outcomes of attention to single or multiple colors using the same paradigm with and without flicker. If application of multiple attentional foci in color space divides the processing resources equally between the two targets, then attentional modulation of SSVEPs elicited by targets should be halved when moving from single to dual targets. Increases in target-distractor proximity should also incur costs in terms of decreased effectiveness of selection—behaviorally, by reduced sensitivity to signal color; neurophysiologically, by SSVEP amplitudes driven by targets being decreased and SSVEP amplitudes driven by distractors being increased. Finally, if operational characteristics of perception and attention are both well captured within the same perceptually uniform color space, equal distance in color space should yield equal effectiveness of attentional selection.

## Materials and methods

### Participants

Twenty-five volunteers in total took part in the study, but five were removed from the final sample. One participant was excluded due to technical problems during the EEG recording. Four other participants were excluded due to inadequate task performance in the EEG session (identified as *d*’s < 0 in any of the conditions). The remaining 20 participants (four male) were on average 23 years old (range 21–27 years). With a sample size of 20 participants and a significance level of 0.05, a paired samples t-test would yield 80% power with an effect size of *d* = 0.66 (calculated with package pwr for R; Champely et al. 2020). In light of previous work in the field, which generally reports large effects of attention on SSVEP amplitudes (>0.8; e.g. Andersen et al. 2008; Andersen et al. 2012) this was deemed to be a sufficiently large sample.

All participants were right-handed and had normal or corrected-to-normal vision and normal color vision as assessed by the Cambridge Color vision test (CCT; Regan et al. 1994). All participants reported having no neurological or psychiatric history and gave written informed consent prior to testing. Participants were recruited amongst the University of Aberdeen students and were reimbursed for their time and effort. The study was approved by the ethics committee of the School of Psychology, University of Aberdeen and was in line with the Declaration of Helsinki (1964).

### Stimuli and procedure

Stimuli were presented on a 21-inch ViewSonic P227f CRT monitor, calibrated using a ColorCAL 2 (CRS, UK) and controlled by a VISaGe (CRS, UK) system. The resolution was set to 640 × 480 pixels with a refresh rate of 120 Hz. CRS toolbox and CRS color toolbox for MATLAB (MathWorks, USA) were used to run the experiment. Color conversions were based on 1931 color matching functions and measurements of the spectra of the monitor phosphors taken by a SpectroCAL (CRS, UK). The behavioral session was done in a dark room and the EEG session in an electrically shielded, sound-attenuated chamber, each with a ViewSonic P227f monitor controlled by a VISaGe system. The viewing distance was ~70 cm. The monitor was the only source of light.

The stimuli consisted of four fully overlapping random dot kinematograms (RDKs) that differed in hue (0°, 180°, 90°, 270°, 45°, and 135°, corresponding to red, green, yellow, blue, orange, and lime; see Table 1) but were of equal chroma and lightness in CIE LCh space (C = 34 and L = 60). Perceptual color spaces are continuous 3D spaces with the dimensions of hue, colorfulness and lightness (e.g. CIELAB, CIELUV; Fairchild 2013). Euclidean distance (ΔE) between two points in these spaces is taken to be a measure of their perceptual color difference, with a just-noticeable difference being equal to a ΔE of 2.3. CIELAB space is used in studies on color-based attention under the assumption that it is fully perceptually uniform and therefore enables researchers to collapse data across individual colors, as long as same color distances between targets and distractors are used (e.g. Stormer and Alvarez 2014). However, while CIELAB does a decent job at representing appearance of colors, it is worth noting that the perceptual unique hues (red, green, yellow, and blue) do not align directly with the CIELAB axes (Fairchild 2013). Assuming illumination equivalent to D65, unique hues lie approximately at CIELAB hue angles of 24° (red), 90° (yellow), 162° (green), and 246° (blue) (Fairchild 1996; for an in-depth analysis, see Seymour 2020). Despite these caveats, CIELAB is well suited for the current study because it provides a space in which relative perceptual distances between hues can be easily controlled, while ensuring that saturation, colorfulness and lightness remain constant across samples (Schiller et al. 2018).

**Table 1:** Stimulus and background color coordinates in CIELCh and CIE 1931.

Color	CIELCh			CIE 1931		
	L	C	h	x	y	Y
Red	60	34	0	0.380	0.297	28.12
Green	60	34	180	0.247	0.361	28.12
Yellow	60	34	90	0.397	0.418	28.12
Blue	60	34	270	0.230	0.242	28.12
Orange	60	34	45	0.425	0.361	28.12
Lime	60	34	135	0.322	0.426	28.12
Background	55	0	0	0.313	0.329	23.00

**Fig. 1:**
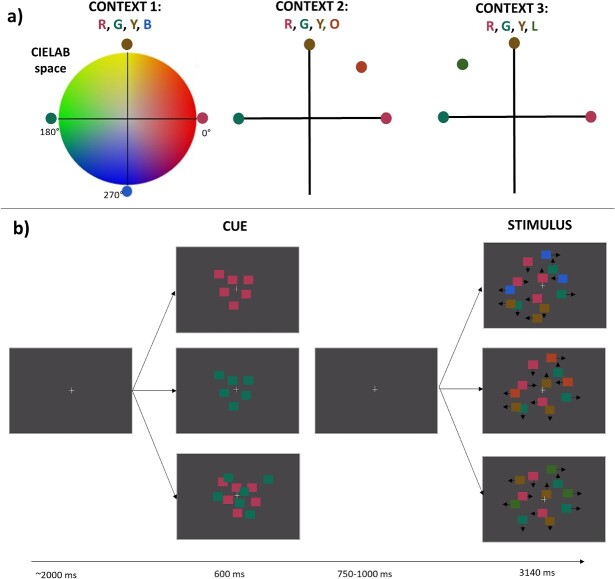
**Stimulus colors and trial outlook.** a) Stimulus colors. Colors were selected from the perceptually uniform CIELAB space (see Table 1 for coordinates) to generate three sets of contexts. Red and/or Green were targets in each context. Yellow was the fixed distractor, also present in each context. There were three context-specific distractor colors: 1) blue, generating a context with equally spaced colors in CIELAB; 2) orange, generating a context in which a distractor was closer to the red target; and 3) lime, generating a context in which a distractor was closer to the green target. b) Trial outlook. Participants were cued to attend either red or green alone or both colors simultaneously. After a delay of 0.75–1.0 s they observed a stimulus interval with the four color RDKs intermingled spatially and moving at random. Each stimulus interval could contain between zero and three brief coherent movements (left, right, up, or down) of either the attended or the unattended colors. In the figure, the number of dots is reduced for the sake of clarity. Colors cannot appear exactly as they did on a calibrated display but have been adjusted to closely resemble their appearance in sRGB space.

The approximate appearance of the stimuli is depicted in Fig. 1, together with the trial outlook. Colors were presented in three color contexts. In the first context, the colors were equally spread in CIELAB space (red, yellow, green and blue). In the second context, we kept the yellow distractor and replaced the blue distractor with orange, to place it closer to red (red, orange, yellow, and green). Lastly, in the third context, the blue distractor was replaced with lime, to place it closer to green (red, yellow, lime, and green). Rather than deploying distractors and targets pooled from different sections of the CIELAB as in Stormer and Alvarez (2014), we opted to evaluate in detail shifts within one quadrant of the color space. This allows us to quantify any hue-specific nonuniformities. Within our design, the yellow distractor served as an important comparison point both between and within color contexts. Meanwhile, the orange and lime distractors are both placed in the same half of hue space but occupy a position nearer to one of the targets (red and green, respectively). The background was metameric to D65 (CIE 1931 coordinates: *x* = 0.3128, *y* = 0.3290, Y = 23 cd/m^2^).

For the EEG session each color was tagged with a specific flicker frequency. The color red flickered at 8.57 Hz; green at 10 Hz; yellow at 12 Hz and depending on the context either blue, orange, or lime at 15 Hz. These frequencies correspond to flicker cycles of 14, 12, 10, and 8 frames, respectively, at our monitor refresh rate of 120 Hz. The on–off ratio for each frequency was 50-50; e.g. 10 Hz flicker was produced by presenting the dots for six frames and then switching them off for another six frames.

While luminance and chromatic mechanisms have different temporal response dynamics, SSVEPs driven by combined color and luminance signals are an outcome of nonlinear summation of the two constituent signals which combines properties of both, with a faster latency than an isoluminant chromatic stimulus but also a higher amplitude than a luminance-isolating stimulus (Martinovic and Andersen 2018). SSVEPs driven by different cone-opponent signals sum differently with luminance contrast, and this summation is also likely to be influenced by temporal frequency. However, when selective attention is deployed to one of two colors, attentional modulation of the said SSVEP signal remains rather stable across different hues, as long as they are all superimposed on equivalent luminance pedestals (Martinovic and Andersen tested 0.37 and 0.71 Weber contrast; in the present study, the luminance pedestal equates to 0.223 Weber contrast). This makes the SSVEP frequency tagging method highly suitable for studying color-based attention. However, the presence of luminance contrast in the stimulus should not be fully neglected when interpreting the results, as it facilitates the performance of the coherent motion detection task. In isoluminant conditions, the coherent motion task would become more difficult and in the presence of relatively fast flicker would likely become even more driven by the L and M-cone related signals picked up by the luminance system (for potential models, see Mullen et al. 2003; Allard and Faubert 2014). In the present study, attentional selection itself remains orthogonal to motion coherence processing: attention needs to target the chromatic component of the stimulus since the luminance component is constant and thus uninformative for target identity (for an extended discussion, see Martinovic and Andersen 2018).

Final methodological consideration concerns the use of a fixed assignment of flicker frequencies to colors, rather than a counterbalanced assignment. Although the absolute magnitude of SSVEP amplitudes differs across colors and across frequencies, we have generally observed equivalent patterns of attentional modulation of color for frequencies in the range employed here (Adamian and Andersen 2023). Thus, any potential benefit of counterbalancing frequencies would not have outweighed the costs in terms of complexity of analysis and reduced precision of measurement, due to the increased number of conditions for each color, but with fewer trials per condition.

The field occupied by the random dot stimuli extended over a circular area with a diameter of 15.642°. Each of the four colors was represented by 75 squares of 0.391° of visual angle, which made 0.061° displacements per frame in a random direction. The squares were drawn in random order to prevent systematic overlap. At the start of each trial a white fixation cross (0.488° by 0.488°) was presented for ~2 s, followed by the presentation of a cue that lasted 600 ms. The cue instructed participants to attend to one color (either red or green) or two colors (both red and green), by presenting a static frame of dots in the to-be-attended color(s). The cue was followed by 0.75-1 s of fixation prior to the motion interval with all four RDKs, which lasted 3.14 s. The motion display also contained the fixation cross and participants were instructed to maintain their gaze at fixation throughout each trial. Target and distractor coherent motion events were embedded within Brownian-like dot motion, with the first 500 ms being event-free. Participants used a CT-6 button box (CRS, UK) and were instructed to respond to coherent motion of the cued color(s), whilst ignoring any such motion from uncued colors. A coherent motion lasted 400 ms and consisted of 50% of the dots from the same color, moving in the same cardinal direction (up, down, left or right). Dots that constituted the 50% coherence motion event were randomly selected on each frame to further prevent any spatial tracking strategy from being effective. The onsets of sequential coherent motion events were separated by at least 700 ms. Responses that occurred within 250–900 ms after the onset of coherent motion events in attended or unattended dots were counted as hits or false alarms, respectively. Similarly, events that did not receive a response in this period were counted as misses or correct rejections. As the task is to attend a specific color for the purpose of coherent motion detection (i.e. participants must select the correct target color to be able to perform the task accurately, once they detect a motion signal), making a false alarm to a distractor-color coherent motion or missing a target-color coherent motion is driven by a lack of sensitivity for color, and thus aligned with the assumptions of the signal detection theory. Participants undergo training prior to the experiment to learn to perform the coherent motion task at ceiling level. Any responses to noncoherent motion would result from noise crossing the decision criterion in the motion signal detection system. Such responses are extremely rare, as participants quickly learn to respond only to coherent motion.

The experiment consisted of two separate sessions: a behavioral and an EEG session. The main differences between them were (i) the inclusion of flicker-frequency tagging and (ii) the reduction in the number of coherent motion events in the EEG session, aimed at effectively capturing attention-related processing using SSVEPs (for a review, see Andersen et al. 2011b). Before the start of the behavioral session, participants were asked to do a practice block of 40 trials. During practice they were provided with feedback sounds: a low beep (600 Hz) corresponded to an incorrect response (false alarm/miss) and a high beep (1500 Hz) indicated a correct response (hit). Prior to the EEG recording, participants did another practice set of 15 trials to familiarize themselves with the now flickering stimuli.

For the behavioral session, participants performed 270 trials, with 30 trials for each of the nine conditions (3 attentional conditions × 3 color contexts). All the trials contained between one and three coherent motion events, resulting in 540 events in total and 60 events per condition (15 per color). This means that each participant was presented with 180 target events (90 per color), 90 red and green distractors (45 per color), 135 yellow distractors, and 135 blue/lime/orange distractors (45 per color). In the EEG session there were a total of 405 trials (45 per condition). In each condition, 30 trials contained no events and 15 trials had between one and three events, for a total of 30 events (i.e. 270 events across the whole experiment). It was not possible to equalize the number of target and distractor events across colors within these parameters so these were assigned randomly, resulting in small differences between the number of events in each color seen by each of the participant (ranges for red and green targets: 81–103 events, red and green as distractors: 35–53, yellow distractors: 53–76, lime/blue/orange distractors: 58–73). These small fluctuations are fully accounted for within the statistical analysis, as generalized linear mixed effect statistical models are fit to the data combined across experiments with experiment as a fixed effect and participant as a random effect.

The completion of the behavioral session, including the color vision test and one practice block took approximately an hour. The EEG session lasted ~2.5 h, with the recording lasting ~1 h and the rest of the time devoted to the brief practice block and electrode setup and removal.

### Experimental design and statistical analysis

#### Behavioral data

Statistical analyses were implemented in R (version 4.2,1, R_Core_Team 2016), using packages tidyverse (version 1.2.1, Wickham et al. 2019), lme4 (version 1.1-31, Bates et al. 2015), effect size (version 0.8.2, Ben-Shachar et al. 2020), emmeans (version 1.8.3, Lenth et al. 2019), performance (version 0.10.1, Lüdecke et al. 2021), DHARMa (version 0.4.6, Hartig 2022) and lmeresampler (version 0.4.2, Loy 2023).

To establish how attentional focus and stimulus context affected participant performance, we analyzed dichotomous outcomes elicited by targets and distractors (i.e. whether events were responded or not responded to). We used generalized linear mixed effect models (GLMMs) on the binomial single-trial data elicited by target or distractor colors, as implemented in the R statistic package lme4. While ordinary logit models have many advantages over ANOVAs on percentage data (e.g. they resolve the potential issue of confidence intervals outside of the 0–100% range), mixed logit models have the further advantage of being able to account for random subject effects (Jaeger 2008). As mentioned earlier, we combined the behavioral data from both experiments prior to fitting the models. Contrasts were set to simple coding: each level of the factor was compared to the reference level, with the intercept set to grand mean. Full details of the best fitting models, including their reference levels, is presented in Supplementary Materials. When fitting GLMMs, maximal random effect structure that was possible was applied, while maintaining goodness of fit (Barr et al. 2013). We verified that the model residuals were normally distributed and did not suffer from overdispersion or excessive outliers using a simulation-based approach implemented in the DHARMa package (Hartig 2022). We also verified that models with multiple predictors did not suffer from predictor collinearity using the check_colinearity function from the *performance* R package on an additive model (Lüdecke et al. 2021).

Models were statistically evaluated by iteratively removing higher-order interactions between fixed effects from the model to verify if it significantly reduced their goodness of fit. In Supplementary Materials we report all estimates of the best-fitting, final models, which include all the fixed effects and interactions that could not be removed. Post-hoc tests on these final models were performed using omnibus paired t-tests on the highest order interactions remaining in the model, corrected for multiple comparisons (*P* < 0.05) with the “mvt” method from emmeans package (Lenth et al. 2019). This method relies on the multivariate t distribution with the same covariance structure as the estimates to determine the *P* value adjustment.

Responses to targets were fitted with a GLMM with fixed effects of *context (blue, orange or lime), attentional focus (focused on a single target or divided between two targets)*, *target color eliciting the response (red or green),* and *experiment (behavioral or EEG).* Responses to distractors were analyzed separately for the fixed distractor (yellow) and the context-dependent distractors (blue, orange and lime). For yellow, the GLMM included the fixed effects of *context (blue, orange, or lime), attentional focus (to red, green, or both targets),* and *experiment (behavioral or EEG).* For blue, orange and lime, the GLMM included the fixed effect of *distractor color (blue, orange, or lime)*, *attentional focus (to red, green or both targets),* and *experiment (behavioral or EEG).* Finally, when red and green were attended on their own, these two colors would also act as distractors. Responses driven by these red and green distractors were also investigated by fitting a GLMM with the fixed effects of *context (blue, orange, or lime)*, *distractor color (red or green),* and *experiment (behavioral or EEG).* This control analysis aimed to verify if the target colors were equally effective when they adopted the role of a distractor. Having selected two colors from the opposite sides of the color space, we expected that this would be the case.

**Fig. 2:**
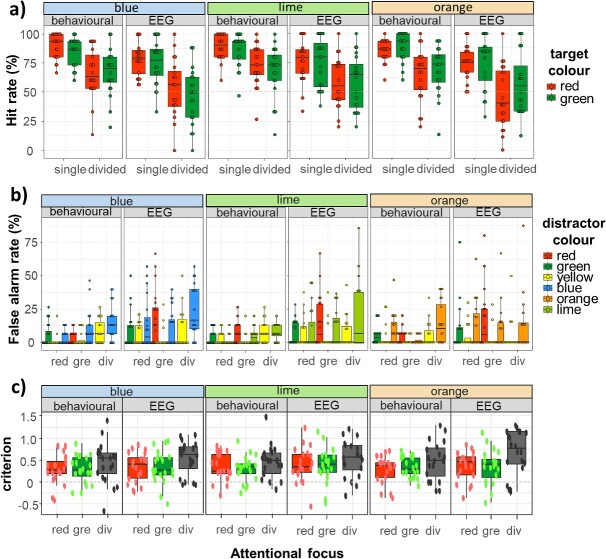
**Box plots of behavioral findings: Hit rates (top panel), False Alarm rates (middle panel) and criteria (bottom panel).** Within each panel, the data is grouped by experiment (behavioral or EEG) as well as context, determined by the following distractors: blue, lime and orange. Attention could be focused on a single target color (i.e. red or green, abbreviated as “gre”) or divided across these two targets (i.e. divided, abbreviated as “div”). To better capture the distribution of the data, we show box plots with individual data points superimposed.

To narrow down the interpretation of target and distractor-related performance, we also analyzed response biases. We used criterion (c) as the most basic measure of response bias, combining false alarms (FAs) across the two distractor colors into a single FA rate prior to the calculation of criterion for red, green or dual targets for each color context. When false alarm and hit rates sum to 100% c = 0, if both are 99% then c = −2.33 and if both equal 1% then c = 2.33 (Macmillan and Creelman 2004). Thus, criterion below 0 reflects a liberal response pattern (FAs exceed misses) and criterion above 0 reflects a conservative response pattern (misses exceed FAs). Prior to calculating the criterion, hit rates of 100% and FA rates of 0% were adjusted according to Wickens’ (2002) proposal, which assumes that should the number of trials have been doubled, at least one hit or FA would have been observed. The criterion is informative on whether attention to multiple targets leads to a change in the position of the “decision axis” about the presence or absence of the signal. The number of target events when attention is divided across two colors increased from 25% to 50% of all coherent motion events, while the number of distractor events fell from 75% to 50%. Target prevalence influences response criterion rather than sensitivity (i.e. *d*’) as it leads to trade-offs between misses and FAs (Wolfe and Van Wert 2010). In Fig. 2a from Wolfe and Van Wert’s (2010)) visual search study, the shift in criterion remains negligible when manipulating target prevalence between 25% and 50%. Nevertheless, quantifying the criterion in addition to the hit and FA rates will allow for a more balanced interpretation of the behavioral findings. We analyzed criterion shifts by fitting a linear mixed effect model (LMM) with the fixed effects of *context (blue, orange or lime), attentional focus (to red, green, or both targets),* and *experiment (behavioral or EEG).*

#### E‌lectroencephalographic recording, processing and analysis

Brain electrical activity was recorded at a sampling rate of 256 Hz from 64 Ag/AgCl electrodes using a Biosemi ActiveTwo EEG system (BioSemi, Netherlands). Lateral eye movements were monitored with a bipolar outer canthus montage (horizontal electrooculogram). Vertical eye movements and blinks were monitored with a bipolar montage positioned below and above the left eye (vertical electrooculogram). During recordings, the CMS (Common Mode Sense) and DRL (Driven Right Leg) electrodes were used to drive the average potential for each participant as close as possible to the AD-box reference.

EEG data were processed using the EEGLab toolbox (Delorme and Makeig, 2004) in combination with custom-made procedures in MATLAB (The MathWorks, Natick, MA). A period of 500 ms after stimulus onset was discarded to exclude the evoked response to stimulation onset and to allow the SSVEP sufficient time to build up. Further, from 2 900 ms after stimulus onset, the data were also discarded, as in this period participants might become aware that no further events were possible, and this knowledge could have led participants' attention to diminish. This resulted in epochs of 2 400 ms duration being extracted for SSVEP analysis. All epochs with target or distractor onsets occurring within the epoch were excluded from the SSVEP analysis. This ensured that the analyzed data were not contaminated by activity related to coherent motion or manual responses and left a total of 30 epochs for each condition. All epochs were detrended (removal of mean and linear trends). Epochs with eye movements or blinks were rejected from further analysis, and all remaining artifacts were corrected or rejected by means of an automated procedure (FASTER; Nolan et al. 2010). The average rejection rate was 12%, leaving an average of 27 trials per condition for analysis (range 14-30). While FASTER was conducted using Fz as reference, data were subsequently transformed to average reference. All epochs within the same condition were averaged for each participant. These means were Fourier-transformed, and SSVEP amplitudes were quantified as the absolute value of the complex Fourier-coefficients at the four stimulation frequencies. Based on the topographical distribution of mean SSVEP amplitudes a cluster of five electrodes (POz, Oz, O1, O2, and Iz) where amplitudes were maximal was chosen and amplitudes were averaged across these electrodes for statistical analysis. Attentional effects were computed by subtracting amplitude when unattended from amplitude when attended (A-U).

To investigate how the attentional increase in SSVEP amplitudes was influenced by the different conditions of the experiment, we fitted linear mixed effect models (LMMs) on the strength of the attentional effect (A-U) with *attentional focus (to a single target or divided between two targets), context (blue, orange, or lime),* and *target color from which responses are analyzed (red or green)* as fixed effects and participant as a random effect. We opted for LMMs as they are robust to nonnormally distributed data and instead require the residuals to be normally distributed (Schielzeth et al. 2020). Since the changeable distractors were physically different (blue, orange, and lime) and thus elicited vastly different amplitudes (consistent with e.g. Martinovic and Andersen 2018; Martinovic et al. 2018), the distractor-driven amplitudes could not be fully compared between contexts. To investigate how distractors were processed between contexts, we therefore focused exclusively on the SSVEP amplitude elicited by yellow, which was the distractor that was constantly present. A LMM analysis with *context (blue, orange, or lime),* and *attentional focus (to red, green, or split)* as fixed effects and participant as a random effect was conducted. We also fitted a LMM with *attentional focus (red, green, or split)* as a fixed effect and participant as a random effect to amplitudes driven by each of the context-specific distractor colors (blue, orange, and lime) separately, to reveal whether their neural processing was affected by the target color(s). The LMMs were run using the same software tools as for the behavioral data, including the same method for conducting assumption checks and pairwise differences for the estimated means.

Finally, to understand the properties of the putative hue-selective foci more fully, we further evaluated differences in amplitudes between single-target and dual-target selection. Our simple model is based on selection of single or multiple confined regions in hue-space and posits that color selection operates through modulating the processing of neurons that respond to the hue(s) within this region. Both the dimension-weighting and relational models of attention agree that attentional settings for color are influenced by their categorical status (Found and Muller 1996), which can be used to set selection weights relative to the other colors in the display (e.g. “redder than” or “yellower than”; Becker 2010). To capture this breadth of target-representation tuning, we assume a relatively wide selection region, encompassing an area of +/− 45° in color space (for similar extent of color tuning in visual working memory, see Bays et al. 2009). A simple mechanistic model, in which a transition from one to two attentional foci would reduce the processing resources in half, would predict that attentional SSVEP modulation should be halved when combining the two attentional settings. This is equivalent to evaluating a mean-amplitude model in which amplitude when selecting two features concurrently is equal to the average of amplitudes when attending single feature values individually (for similar findings from orientation and color, see Andersen et al. 2008; Andersen et al. 2015). The mathematical equivalence of these statistical tests stems from the fact that attentional increases when attending red alone or together with green are calculated by subtracting the same unattended amplitude (i.e. red when green is attended). Thus, despite the values being different, the statistical comparison relies on the identical underlying data (i.e. testing if (AmpR_,r_ + AmpR_g_)/2 = AmpDiv_r_ vs. (AmpR_r_—AmpR_g_)/2 = AmpDiv_r_—AmpR_g_ where AmpR_r_ is amplitude for red when attending red, AmpR_g_ is amplitude for red when attending green and AmpDiv_r_ is amplitude for red when dividing attention). These predictions were evaluated by solving for the multiplier *p* in the following equations: AmpDiv_r_ = *p* * (AmpR_,r_ + AmpR_g_) and AmpDiv_g_ = *p* * (AmpG_g_ + AmpG_r_). We then compared the difference between the obtained values and 0.5, to evaluate the accuracy of our predictions.

## Results

### Behavioral findings

For the behavioral experiment, collapsing the data across all stimuli and conditions yields hit rates of 78% ± 12%, false alarm rates of 6% ± 5%, with a criterion of 0.36 ± 0.27 (means and standard deviations). In the EEG experiment, hit rates were 65% ± 13%; false alarm rates 9% ± 10% and criterion 0.43 ± 0.31 (means and standard deviations; see Fig. 2). Median number of button presses occurring when no coherent motion event was presented was very low, at 3 per participant across the duration of the EEG experiment (range 0–22).

This shows that the motion detection task is challenging but achievable, reflected by the high percentage of hits and the low rate of false alarms. A positive but relatively small criterion value indicates that participants adopted a somewhat conservative strategy. Once again this confirms that the task was achievable. The small decrease in overall hit rates and increase in false alarm rates in the EEG experiment can be attributed to the addition of flicker. Some participants particularly struggled performing the motion detection task on flickering RDKs: as described in the Participant section, four observers became unable to perform the task reliably and were removed from the sample.

Target-elicited responses were analyzed using a GLMM with the following fixed effects—*context (blue, orange, or lime), attentional focus (focused on a single target or split between two targets)*, *target color (red or green),* and *experiment (behavioral or EEG)*. We also added random by-participant intercepts, to account for more basic differences in overall performance. The best-fitting model included attentional focus (χ^2^(1) = 312.39, *P* < 0.001), experiment (χ^2^(1) = 113.18, *P* < 0.001) and an interaction between target color and context (χ^2^(2) = 8.964, *P* = 0.011; for full details of this model, see Supplementary Materials). As expected, participants were considerably more accurate in detecting target events when they attended a single color as opposed to when they were asked to divide attention between two targets (OR 3.22, 95% CI 2.81-3.68). The odds of target detection were also substantially increased in the behavioral experiment (OR 2.06, 95% CI 1.8-2.35), indicating interference of stimulus flicker with motion processing. Finally, post-hoc pairwise comparisons of the target color by context interaction revealed that hit rates for red in the lime context tended to be higher than in the orange context (OR 1.435, 95% CI 1.052—1.96, *Z* = 3.157, *P* = 0.013). None of the other pairwise comparisons between contexts reached significance (all *P*s > 0.343). To summarize these findings, a major determinant for hit rates appears to be whether attention is focused or divided, with a large cost in performance when attention is split between the two targets. This is consistent with a decreased efficiency of target enhancement. Such a cost could stem from the need to re-allocate central, top-down resources to the monitoring of a secondary attentional template or the continuous need to switch resources between two attentional templates, as they cannot be monitored concurrently. There is also an increase in detection of red targets in the evidently less distracting lime/yellow context compared to the more distracting orange/yellow context, with hit rates being similar across the other contexts.

Responses to yellow distractors were analyzed using a GLMM with fixed effects of *context (blue, orange, or lime), attentional focus (to red, green or both targets),* and *experiment (behavioral or EEG),* as well as random by-participant intercepts*.* The best-fitting, final model included an interaction between attentional focus and experiment (χ^2^(2) = 6.325, *P* = 0.042), with no contribution of context (see Supplementary Materials for detail). Thus, the analysis confirms that the yellow distractor was contextually neutral. We also found some unexpected differences between experiments. Division of attention led to higher false alarms in the behavioral experiment (red vs. divided: OR 0.318, 95% CI 0.163-0.620, *Z* = −4.465, *P* < 0.001; green vs. divided: OR 0.232, 95% CI 0.110-0.489, *Z* = −5.309, *P* < 0.001), with similar performance across the two foci (red vs. green: OR 1.370, 95% CI 0.574-3.270, *Z* = 0.982, *P* = 0.891). However, in the EEG experiment, division of attention increased the odds of yellow-driven false alarms when compared to the green focus (OR 0.375, 95% CI 0.145-0.968, *Z* = −2.801, *P* = 0.039) but not for red focus (OR 0.813, 95% CI 0.381-1.733, *Z* = −0.741, *P* = 0.965; red and green foci did not statistically differ, OR 2.169, 95% CI 0.833-5.649, *Z* = 2.192, *P* = 0.184). As red and green also acted as distractors when attention had a single focus (i.e. when red was attended, green acted as distractor, and vice versa) we fitted a GLMM with factors of *context* (blue, orange and lime), *distractor color* (red, green), and *experiment (behavioral or EEG)* as well as random by-participant intercepts to assess any potential differences. As for yellow distractors, we found an interaction between attentional focus and experiment (χ^2^(1) = 6.874, *P* = 0.009; see Supplementary Materials for full statistical details), with no differences between the two distractors in the behavioral experiment (OR 1.00, 95% CI 0.583-1.715; *Z* = 0.00, *P* = 1.00) but fewer false alarms to green relative to red in the EEG experiment (OR 0.44, 95% CI 0.256-0.755, *Z* = 3.732, *P* < 0.001). This is consistent with lower efficiency of processing red coherent motion events in the EEG experiment and likely to stem from the reduction in motion salience as red flickered at the lowest frequency (8.57 Hz).

For the crucial, context-dependent distractor, there was a significant interaction between all three fixed effects: attentional focus, context and experiment (χ^2^(4) = 21.377, *P* < 0.001; see Supplementary Materials). The interaction between focus and context was predicted—we decomposed the statistical effects by comparing these factors within each experiment. In the behavioral experiment, attending to red as opposed to dividing attention between the two targets led to a decrease in blue false alarms (OR 0.198, 95% CI 0.064-0.609, *Z* = 4.609, *P* < 0.001) without robust differences in false alarm rates between focusing on red vs. green (OR 0.314, 95% CI 0.098-1.009, *Z* = 3.175, *P* = 0.051) and dividing attention vs. focusing on green (OR 0.630, 95% CI 0.276-1.435, Z = 1.795, *P* = 0.848). Attending red as opposed to dividing attention also led to a decrease in false alarms to lime (OR 0.155, 95% CI 0.027-0.895, Z = −3.401, *P* = 0.024) without robust differences in false alarm rates between focusing on red vs. green (OR 0.269, 95% CI 0.043-1.680, Z = −2.293, *P* = 0.471) and divided attention vs. green (OR 0.576, 95% CI 0.186-1.786, Z = −1.559, *P* = 0.948). Finally, there was a large increase in orange false alarms when attending red compared to green (OR 6.446, 95% CI 1.328-31.29, *Z* = 3.772, *P* = 0.007), while attending green as opposed to dividing attention between the two targets led to a decrease in orange false alarms (OR 0.089, 95% CI 0.019-0.415, Z = 5.023, *P* < 0.001; no robust differences for red vs. dividing attention, OR 0.575, 95% CI 0.246-1.341, *Z* = 2.091, *P* = 0.637). Therefore, distraction in divided attention is consistently increased relative to the single focus for the more distal target color (green target for orange distractor; red target for lime and blue distractors). In addition to that, increased false alarms elicited by the blue distractor when attentional focus included green indicate that in CIELAB, blue is attentionally more proximal to green than to red, invalidating our original assumption that yellow and blue distractors would both provide a supposedly neutral context. On the other hand, there were no robust differences between blue, lime or orange distractors in the EEG experiment (all *P*s > 0.075). To understand this discrepancy, it is necessary to look at between-experiment differences. The behavioral experiment had fewer false alarms relative to the EEG experiment but these differences were not uniform. For blue distractors, there were fewer false alarms only for red focus (OR 0.223, 95% CI 0.0634-0.782, *Z* = −3.825, *P* = 0.005, otherwise *P* > 0.983) and for orange distractors, fewer false alarms were observed for the green focus (OR 0.129, 95% CI 0.024-0.686, *Z* = −3.918, *P* = 0.003). Thus, it appears that flicker led to the elevation of false alarm rates in what would have otherwise been a relatively nonchallenging condition (compare behavioral and EEG panels in Fig. 2), obfuscating previously robust between-condition differences. This further supports our choice to perform both a behavioral and an EEG experiment, with the intention to obtain more valid coherent motion detection data in the absence of flicker.

Finally, criteria (Fig. 2, bottom panel) were fitted with a LMM with fixed effects of *context* (blue, orange, and lime), *attentional focus* (red, green, both), and *experiment* (behavioral and EEG), as well as random effect of by-participant intercepts. The best fitting model included fixed effects of *attentional focus* (χ^2^(2) = 32.022, *P* < 0.001) and experiment (χ^2^(1) = 5.224, *P* = 0.022, with EEG experiment being more conservative by 0.072 ± 0.030 SE; for full statistical detail, see Supplementary Materials). A more conservative criterion also manifested itself in the divided attention condition in comparison to attending to red alone (effect size = −0.182 ± 0.037 SE, t(343) = −4.969, *P* < 0.001) or green alone (effect size = −0.184 ± 0.037 SE, t(343) = −5.023, *P* < 0.001). There was no difference in criteria between the two single foci (effect size = 0.002 ± 0.037 SE, t(343) = 0.054, *P* = 0.998). A more conservative criterion for divided attention indicates that the associated increase in false alarms was not due to an adoption of a more liberal response criterion. This is consistent with our observation that divided attention also leads to a large fall in hit rates when compared to focused attention.

In summary, our behavioral data reveals three key patterns: (i) we confirm that flicker selectively decreases the saliency of motion signals for stimuli at lower temporal rates when compared to stimuli at higher temporal rates (Nakayama and Silverman 1984), complicating the comparison of behavioral data for flickering stimuli; (ii) we find that divided attention leads to a large reduction of hit rates and a comparatively smaller increase in false alarm rates, as corroborated by the more conservative response criterion; (iii) as predicted, false alarms are affected by context, but the assumption that equivalent coordinates in CIELAB space translate to equal attentional selection is not supported. Lime distractors seem less potent in eliciting false alarms than orange distractors, despite being fully matched to them in CIELAB. Additionally, the supposedly neutral blue distractors cause less distraction for red as opposed to green targets.

### E‌lectroencephalographic experiment: Steady-state visual evoked potential results

**Fig. 3:**
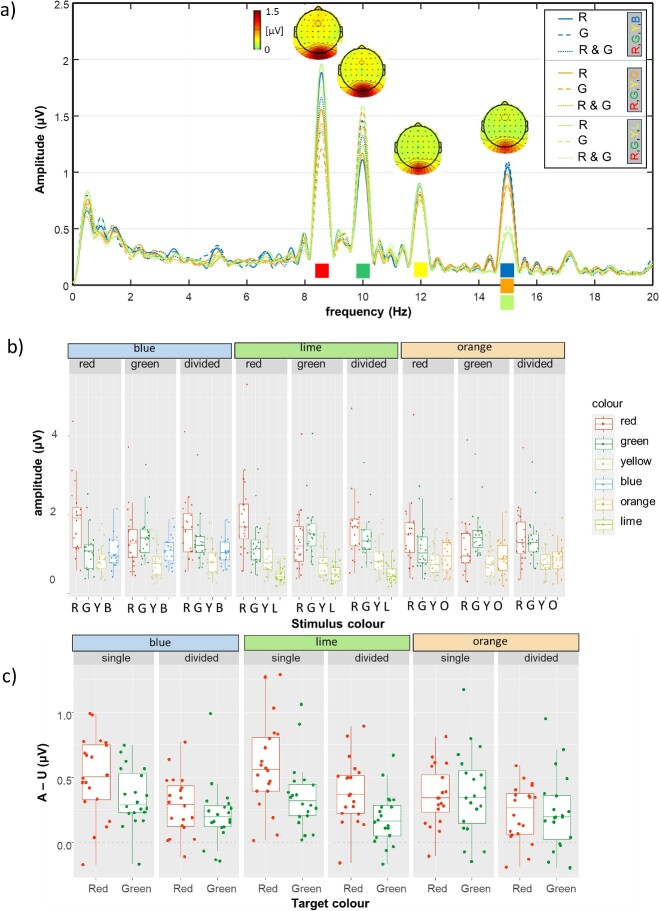
**SSVEP results**. a) Spectra and topographies. Full lines depict amplitudes elicited when red was the target color, dashed lines depict amplitudes elicited when green was the target color and dotted lines depict amplitudes when attention was divided between red and green. The color of the line depicts the context, which in addition to red, green and yellow included either blue, orange or lime. b) Box plot of SSVEP amplitudes, with individual data points overlaid on the graph. c) Box plot of attentional effects calculated from SSVEP amplitudes elicited by green and red targets. Unattended amplitude for the red color is taken from the “attend green” condition, and vice versa.

SSVEP spectra, topographies and amplitudes are depicted in Fig. 3a-b.

To investigate target enhancement, LMM was fitted to the calculated attentional effects (attended minus unattended SSVEP amplitude; Fig. 3c), with *context (blue, orange, or lime), attentional focus (to a single target or divided between two targets),* and *target color from which responses are analyzed (red or green)* as fixed effects and participants as random effects (see Supplementary Materials for full details of the best fitting model). *Attentional focus* had the biggest influence on SSVEP attentional modulation (χ^2^(1) = 31.318, *P* < 0.001), with an average loss of ~50% (i.e. 0.169 μV) when dividing attention. *Context* and *target color* interacted (χ^2^(2) = 11.005, *P* = 0.004), qualifying the main effects observed for each of these factors individually. Further examination of this interaction revealed that the attentional effect for red in the lime context was higher when compared to red in the orange context (estimate = 0.202 μV, t(226) = 3.956, *P* = 0.001) but not the blue context (estimate = 0.096 μV, t(226) = 1.881, *P* = 0.417); orange and blue contexts did not differ robustly either (estimate = 0.106 μV, t(226) = 2.075, *P* = 0.3044). Meanwhile, attentional effects for green remained relatively stable, not robustly affected by any of the contexts (all ts < 0.718, *P*s > 0.980). Thus, higher target enhancement for red was possible in the presence of yellow and lime. We also performed the statistical analysis separately on red and green data, to avoid any confounds due to comparing data stemming from two stimulation frequencies within the same model. This analysis confirmed all the effects reported above (i.e. for red, there were main effects of both context and focus, while green amplitude was only affected by focus; see Supplementary Materials for statistical tables).

To investigate how distractors were processed between contexts, we first focused on the SSVEP amplitude elicited by yellow, which was the distractor that was constantly present (see Fig. 3b). A LMM with *context (blue, orange, or lime),* and *attentional focus (to red, green or divided)* as main effects and participants as random effects was fitted (full statistical details are in the Supplementary Materials). The best fitting model contained the additive effects of *context* and *attentional focus*. Amplitude for yellow was lower in the orange context (*P* = 0.003), with similar amplitudes across blue and lime contexts (*P* = 0.928). In addition, amplitude was lower when attention was focused on green, compared to when attention was focused on red (*P* < 0.001; red vs. divided attention not significantly different, *P* = 0.987).

Note that in Fig. 3a-b amplitudes elicited by blue, orange and lime at 15 Hz are markedly different. Therefore, we fitted LME models with *attentional focus (red, green, or divided)* as a fixed effect and participants as random effects for each of the changeable distractor colors separately, intending to reveal whether their neural processing was affected by the target color(s). For orange, attentional focus contributed significantly to the model (χ^2^(2) = 5.587, *P* = 0.007), with higher amplitudes when attending red compared to attending green (t(42.1) = 2.821, *P* = 0.0195) or dividing attention between red and green (t(42.1) = 2.822, *P* = 0.0194; green vs. divided attention, t(42.1) = 0.001, *P* = 1.00). For blue and lime, attentional focus did not play a significant role in explaining any of the variance.

In a final analysis, we evaluated if target-related attentional increases at the neural level follow a simple resource allocation model. If this were the case, attentional modulation in divided attention conditions should be half of its magnitude when only single colors are attended (Adamian et al. 2019; Adamian and Andersen 2022; Andersen et al. 2009). Alternatively, if these were implemented independently for each color, akin to selection of feature values from different dimensions (Andersen et al. 2015; Adamian et al. 2019), attentional modulation in the divided attention condition would be as large as in the attend single color conditions. Predictions based on simple resource allocation (i.e. 50% of attentional increase when dividing attention) seem to fit the data extremely well, only slightly underestimating the actual attentional modulation of SSVEP amplitudes. The multiplier *p*, reflecting the reduction in attentional modulation between single and divided attentional foci (AmpDiv_colour_ = p * (AmpColour_red_ + AmpColour_green_), ranged between 0.511 and 0.538, indicating that attentional resources are approximately halved between the two colors (Red, blue context: 0.516, 95% CI 0.486—0.545, t(19) = 1.125, *P* = 0.275; Green, blue context: 0.522, 95% CI 0.492—0.552, t(19) = 1.563, *P* = 0.135; Red, orange context: 0.536, 95% CI 0.500—0.572, t(19) = 2.099, *P* = 0.0494; Green, orange context: 0.520, 95% CI 0.477—0.564, t(19) = 0.983, *P* = 0.338; Red, lime context: 0.538, 95% CI 0.514—0.562, t(19) = 3.35, *P* = 0.003; Green, lime context: 0.511, 95% CI 0.483—0.540, t(19) = 0.843, *P* = 0.41). Summation coefficient for red targets in orange and lime contexts is statistically significantly above 0.5, but this deviation is very small (~4%).

## Discussion

We investigated whether concurrent selection of two color targets can be conceived as a process in which two attentional foci are applied within the multidimensional color space, enacting control on the distribution of processing resources across color representations on the basis of their proximity within this space. To achieve this, we designed an experiment that manipulated both the number of color targets (one or two) and the proximity of distractors to the targets in a perceptual color space (intermediate or closer to one of the targets). In a sustained multicolored RDK coherent motion detection task, we placed red and green targets in one of three contexts: (i) a neutral context with intermediate distractors, falling between the two target hues in CIELAB space (blue and yellow); (ii) a context with one distractor closer to red (orange and yellow); (iii) a context with one distractor closer to green (lime and yellow). In the behavioral experiment, we found that hit rates were overwhelmingly dependent on whether attention was focused or divided, confirming that concurrent selection of two colors was possible, but came at a significant cost to the efficiency of target enhancement. On the other hand, false alarms were more strongly affected by context: blue, orange, and lime distractors affected the performance differentially. In line with this, target-driven SSVEP data were consistent with a combination of two separate foci that controlled the distribution of attention across elements in the scene, with allocated resources being approximately halved with divided attention. On the other hand, representational proximity between targets and distractors affected distractor-elicited neural responses to a much lesser degree compared to behavioral responses. This might indicate that concurrent selection of two categorically distinct colors is mainly limited by a central, resource-related bottleneck, with more minor contributions from a local, perceptual-distance-related bottleneck.

As predicted, we found higher rates of responses to distractors in contexts that put them closer to one color, e.g. orange elicits more false alarms when the target is red and lime elicits more false alarms when the target is green. However, contrary to our predictions, this did not occur evenly across our contexts, which were presumed to be either neutral (blue), closer to red (orange) or closer to green (lime). Blue was found to elicit more false alarms when the target was green, while the overall number of false alarms was the lowest (i.e. most “neutral”) in the yellow/lime context. This unexpected finding implies that while CIELAB may be perceptually uniform, this does not guarantee in any way its uniformity in terms of attentional effects or categorical representativeness of different hues. Unlike simple color discrimination (for a discussion, see Martinovic et al. 2020), performance in a visual search task for color targets is influenced by categorical status (Found and Muller 1996; for an overview, see Martinovic and Hardman 2016). Categories can also be used to set attentional guidance based on an element’s position in relation to the other colors in the display (e.g. “redder than” or “yellower than,” Becker 2010; such relational color selection is likely to be an aspect of good-enough attentional guidance, Yu et al. 2023). We observed higher levels of distractor interference, both behaviorally and neutrally, for the orange color as compared to the lime color. This asymmetry is not consistent with positions of our stimulus colors in CIELAB nor with their positions in relation to unique hues, but is consistent with categorical labelling data, which indicates a larger overlap between orange and red (Malkoc et al. 2005, Fig. 1). The fact that CIELAB may be perceptually but not attentionally uniform poses problems for studies that probe multiple locations in CIELAB but subsequently collapse the data across hues (e.g. Stormer and Alvarez 2014).

In a sustained task such as ours, proximity to categorical best-exemplars may act directly, by facilitating the efficient setting and maintenance of attentional templates (Bae et al. 2015), or indirectly, by facilitating grouping of more similar hues together, be they targets or distractors (Matera et al. 2020). Since the orange distractor is more similar to red than to yellow, this may lead to a spread of activation in accordance with the feature similarity gain model (Martinez-Trujillo and Treue 2004; Oxner et al. 2023), making it more difficult to allocate top-down resources (i.e. to successfully bias competition) towards the red target whilst ignoring orange. On the other hand, if the “yellowness” of the lime distractor can be successfully used to group it together with yellow and away from green, this would make it easier to bias competition towards the green target. Grouping of local motion signals by color appears to precede global motion processing and is under attentional control (Martinovic et al. 2009; Wuerger et al. 2011). If color similarity increases the grouping of colors in the RDK, this would create predicted costs when they need to be selected or ignored independently (e.g. red/orange) but also produce unexpected benefits when they need to be ignored together (e.g. yellow/lime). In fact, the special status of color as an extremely salient feature dimension in terms of attention (see Found and Muller 1996) is at least partly derived by our ability to maintain and use multiple templates in short-term memory that are based on a color’s categorical status, mediated through ease of verbal labelling (Forsberg et al. 2019). As discussed in a recent review, this multidimensionality of color is likely to have important repercussions for color-based attentional selection (Liesefeld and Müller 2019).

In a recent combined SSVEP-fMRI study, attentional color modulations were mainly observed in area V4v (Boylan et al. 2023). Together with VO1 and VO2, this is the area in which color selection has maximal effects (Thayer and Sprague 2023). In V4, color representations are organized in a way that follows the perceptual hue circle but also exhibits categorical clustering (Brouwer and Heeger 2009, 2013). A model has been proposed that attributes this clustering to a color-specific gain change: the gain of each neuron changes as a function of its selectivity relative to the centers of the color categories. If attentional selection in extra-striate cortices is implemented through stimulus–stimulus interactions (for a discussion of this model, see Parker 2020), then attentional modulations would be more effective if aligned with category centers, both in terms of target enhancement and distractor suppression. This is a testable prediction which can be directly examined in future studies, manipulating category representativeness of targets and distractors systematically.

Returning to the highly congruent effects observed in behavioral and neural data elicited by targets, the ~20–25% cost in hit rates for divided attention corresponded to a ~50% reduction in amplitude that was relatively well predicted by a simple averaging of the amplitudes elicited by red and green in the two focused attention conditions. Processing is altered not only for targets, but also for some distractors: orange, which elicited highest behavioral interference when red was attended, also had higher amplitude if the focus of attention was on red alone. However, the behavioral costs were comparatively smaller than for targets, and neural changes were even more limited—9% change in amplitude for orange distractors and no discernible neural changes for lime or blue distractors, despite increased false alarms. While attentional modulations of sensory coding and behavioral readout are both likely to have some neural correlates in V4 (Parker 2020), behavioral readout would also be influenced by multiple further components of processing beyond mere competitive interactions in visual cortices (e.g. the dorsal frontoparietal network, Liu 2019). This implies that the need to maintain two active target templates poses higher costs at later stages of attentional processing. Finally, we observe that orange-driven amplitude is similarly reduced when attentional focus is on green or divided between red and green. Thus, it appears that having a green target template is sufficient to eliminate the allocation of visuo-cortical resources to orange that stems from the adoption of a red target template. In the context of limited parallel or limited serial models of multiple-target selection (Ort and Olivers 2020), serial switching between the two templates during selection would mean that the spreading of perceptual resources to orange should be halved, rather than eliminated. Future studies should attempt to precisely quantify the residual resources allocated to orange whilst attending to red, to provide more decisive evidence disambiguating between parallel (already favored in the literature) or serial selection models.

## Conclusions

Putting these findings together, we have shown that target enhancement in divided attention to color can be predicted from single color selection. Color distances in CIELAB space only partially account for our results, both in terms of behavioral and neural data. The findings of our study pave the way for a more refined exploration of the relations between categorical status, color content (e.g. as identified by hue scaling) and perceptual differences. Should full understanding of these relations be obtained, it may be possible to quantify an attentional color space, which could be used to predict the ease with which colors can be selected together or filtered out as distractors.

## Supplementary Material

supplementary_materials_for_v11_bhae240

## Data Availability

Raw behavioral and averaged EEG data, as well as statistical analysis scripts in R are publicly available on Open Science Framework (OSF): https://osf.io/2kjsx/.

## Notes

*Conflict of interest statement*: The authors state no conflict of interest. For the purpose of open access, the authors have applied a Creative Commons Attribution (CC BY) license to any Author Accepted Manuscript version arising from this submission.
