# Supplementary material for: Division and spreading of attention across color
Source: Cereb Cortex. 2024 Jun 10;34(6):bhae240. doi: 10.1093/cercor/bhae240 (PMC11164655; doi:10.1093/cercor/bhae240)
Supplement: supplementary_materials_for_v11_bhae240 [file supplementary_materials_for_v11_bhae240.docx]

**Supplementary Materials**

**Supplementary Material – Best fitting statistical models**

The tables present estimated parameters, their standard errors or confidence intervals, z-values, p-values and effect sizes for the best fitting GLMM or LMM.

**1 – Behavioural results**

**Table S1** ***GLMM - Responses to target events*.** Reference levels were set to single focus, red target, blue context and behavioural experiment.

Model equation: response ~ 1 + target_colour + attentional_focus + colour_context + experiment + target_colour : colour_context + (1|participant)

| **Fixed Effects** | **Estimate ± SE** | **z value** | **p value** | **Odds ratio, (95% CI)** |
| --- | --- | --- | --- | --- |
| Intercept | 1.057 ± 0.133 | 7.931 | <.001*** | 2.88  (2.22 – 3.74) |
| Green vs. red | -0.014 ± 0.066 | -0.209 | 0.834 | 0.99  (0.87 - 1.12) |
| Divided vs. single | -1.169 ± 0.069 | -17.061 | <.001*** | 0.31  (0.27 - 0.36) |
| Lime vs. blue | 0.139 ± 0.081 | 1.719 | 0.086 | 1.15  (0.98 - 1.35) |
| Orange vs. blue | -0.009 ± 0.080 | -0.108 | 0.914 | 0.99  (0.85 - 1.16) |
| EEG vs. behavioural experiment | -0.721 ± 0.068 | -10.657 | <.001*** | 0.49  (0.43 – 0.56) |
| Difference between red and green targets in lime vs. blue context | -0.022 ± 0.162 | -0.134 | 0.893 | 0.98  (0.71, 1.34) |
| Difference between red and green targets in orange vs. blue context | 0.404 ± 0.160 | 2.529 | 0.011 | 1.50  (1.10, 2.05) |

Random effects: The maximal model that could be adequately fitted to the dataset included random by-participant intercepts, accounting for 0.330 ± 0.574 variance (Estimate ± SD). Residual variance, σ^2^ = 3.29. Interclass correlation coefficient (ICC) = 0.09. There were 5409 responses analysed (3600 from behavioural and 1809 from the EEG experiment) from 20 participants. Conditional r^2^: 0.197; Marginal r^2^: 0.117.

**Notes on model evaluation**: The four-way interaction did not contribute significantly to the model (χ^2^(2) = 2.532, p = 0.282), so it was removed; the same was the case for three-way interactions between attentional focus, context and target colour (χ^2^(2) = 0.268, p = 0.875), context, target colour and experiment (χ^2^(2) = 0.521, p = 0.771), attentional focus, target colour and experiment (χ^2^(1) = 0.105, p = 0.746), attentional focus, context and experiment (χ^2^(2) = 0.356, p = 0.837), and two-way interactions of context by experiment (χ^2^(2) = 0.055, p = 0.973), target colour and experiment (χ^2^(1) = 0.415, p = 0.520), attentional focus and experiment (χ^2^(1) = 1.327, p = 0.249), attentional focus and target colougr (χ^2^(1) = 1.408, p = 0.235), and attentional focus and context (χ^2^(2) = 3.049, p = 0.218).

**Table S2**. ***GLMM - Responses to yellow distractors*.** Reference levels were set to red focus and behavioural experiment.

Model equation: response ~ 1 + target_colour + attentional_focus + experiment + target_colour : experiment + (1|participant)

| **Fixed Effects** | **Estimate ± SE** | **z value** | **p value** | **Odds ratio, (95% CI)** |
| --- | --- | --- | --- | --- |
| Intercept | -3.551 ± 0.234 | -15.185 | <.001*** | 0.03  (0.02 - 0.05) |
| Green vs. red | -0.545 ± 0.239 | -2.282 | 0.022 | 0.58  (0.36 - 0.93) |
| Divided vs. red | 0.677 ± 0.187 | 3.629 | <.001*** | 1.97  (1.37 - 2.84) |
| EEG vs. behavioural experiment | 0.382 ± 0.177 | 2.158 | 0.031 | 1.47  (1.04 – 2.07) |
| Green vs. red / EEG vs. behavioural experiment | -0.459 ± 0.477 | -0.962 | 0.336 | 0.63  (0.25 – 1.61) |
| Divided vs. red / EEG vs. behavioural experiment | -0.940 ± 0.373 | -2.522 | 0.012 | 0.39  (0.19 – 0.81) |

Random effects: The maximal model that could be adequately fitted to the dataset included random by-participant intercepts, which accounted for 0.829 ± 0.911 in variance (Estimate ± SD). Residual variance 3.29 (ICC = 0.20). There were 4042 responses analysed (2700 from the behavioural and 1342 from the EEG experiment), from a total of 20 participants. Conditional r^2^: 0.265; Marginal r^2^: 0.079.

**Note**: The three-way interaction did not contribute significantly to the model (χ^2^(2) = 2.5318, p = 0.282), so it was removed; the same was the case for two-way interactions between attentional focus and context (χ^2^(4) = 4.415, p = 0.353), context and experiment (χ^2^(2) = 0.259, p = 0.879), and the fixed effect of context (χ^2^(2) = 2.384, p = 0.304).

**Table S3**. ***GLMM - Responses to red and green distractors*.** Reference levels were set to red colour and behavioural experiment.

Model equation: response ~ 1 + target_colour + experiment + target_colour : experiment + (1|participant)

| **Fixed Effects** | **Estimate ± SE** | **z value** | **p value** | **Odds ratio, (95% CI)** |
| --- | --- | --- | --- | --- |
| Intercept | -2.787 ± 0.219 | -12.76 | <.001*** | 0.06  (0.04, 0.09) |
| Green vs. red colour | 0.411 ± 0.156 | 2.641 | 0.008 | 1.51  (1.11-2.05) |
| EEG vs. behavioural experiment | 1.030 ± 0.156 | 6.593 | <.001*** | 2.80  (2.06-3.80) |
| Green vs. red across EEG vs. behavioural experiment | 0.822 ± 0.311 | 2.641 | 0.008 | 2.27  (1.24 – 4.19) |

Random effects: The maximal model that could be adequately fitted to the dataset included random by-participant intercepts, which accounted for 0.763 ± 0.873 in variance (Estimate ± SD). Residual variance 3.29 (ICC = 0.19). There were 2689 responses analysed, from a total of 20 participants. Conditional r^2^: 0.243; Marginal r^2^: 0.067.

**Note**: The three-way interaction did not contribute significantly to the model (χ^2^(2) = 1.415, p = 0.493), so it was removed; the same was the case for attentional focus by context (χ^2^(2) = 3.497, p = 0.174) context by experiment (χ^2^(2) = 2.152, p = 0.341) and context (χ^2^(2) = 0.148, p = 0.929).

**Table S4**. ***Responses to blue, orange and lime distractors*.** Reference levels were set to red focus, blue distractor and behavioural experiment.

Model equation: response ~ 1 + attentional_focus + colour_context + experiment + attentional_focus:colour_context + attentional_focus:experiment + attentional_focus:colour_context:experiment + (1|participant)

| **Fixed Effects** | **Estimate ± SE** | **z value** | **p value** | **Odds ratio, (95% CI)** |
| --- | --- | --- | --- | --- |
| Intercept | -2.656 ± 0.225 | -11.810 | <.001*** | 0.07  (0.05, 0.11) |
| Green vs. red focus | 0.036 ± 0.177 | 0.204 | 0.839 | 1.04  (0.73, 1.47) |
| Divided vs. red focus | 0.940 ± 0.154 | 6.098 | <.001*** | 2.56  (1.89, 3.46) |
| lime vs. blue | -0.622 ± 0.160 | -3.892 | <.001*** | 0.54  (0.39, 0.73) |
| orange vs. blue | -0.318 ± 0.150 | -2.118 | 0.034 | 0.73  (0.54, 0.98) |
| EEG expt vs. behavioural expt | 0.947 ± 0.131 | 7.223 | <.001*** | 2.58  (1.99, 3.33) |
| Difference between green and red focus for lime vs. blue | 0.272 ± 0.432 | 0.630 | 0.529 | 1.31  (0.56, 3.06) |
| Difference between divided and red focus for lime vs. blue | 0.156 ± 0.405 | 0.386 | 0.700 | 1.17  (0.53, 2.59) |
| Difference between green and red focus for orange vs. blue | -1.482 ± 0.402 | -3.683 | <.001*** | 0.23  (0.10 – 0.50) |
| Difference between divided and red focus for orange vs. blue | -0.946 ± 0.325 | -2.909 | 0.004** | 0.39  (0.21, 0.73) |
| Difference between green and red focus for EEG vs. behavioural expt | -0.334 ± 0.353 | -0.946 | 0.344 | 0.72  (0.36, 1.43) |
| Difference between divided and red focus for EEG vs. behavioural expt | -0.814 ± 0.308 | -2.645 | 0.008** | 0.44  (0.24-0.81) |
| Difference between lime and blue context for EEG vs. behavioural expt | -0.599 ± 0.319 | 1.874 | 0.061 | 1.82  (0.97, 3.40) |
| Difference between orange and blue context for EEG vs. behavioural expt | 0.013 ± 0.300 | 0.042 | 0.966 | 1.01  (0.56, 1.83) |
| Green vs. red/ lime vs. blue/ EEG vs. behavioural expt | 0.238 ± 0.863 | 0.275 | 0.783 | 1.27  (0.23, 6.89) |
| Divided vs. red/ lime vs. blue/ EEG vs. behavioural expt | -0.173 ± 0.809 | -0.214 | 0.831 | 0.84  (0.17, 4.11) |
| Green vs. red/ orange vs. blue/ EEG vs. behavioural expt | 3.083 ± 0.804 | 3.834 | <.001*** | 21.82  (4.51, 105.46) |
| Divided vs. red/ orange vs. lime/ EEG vs. behavioural expt | 0.244 ± 0.650 | 0.376 | 0.707 | 1.28  (0.36-4.56) |

Random effects: The maximal model that could be adequately fitted to the dataset included random by-participant intercepts, which accounted for 0.883 ± 0.940 in variance (Estimate ± SD). Residual variance 3.29 (ICC = 0.21). There were 4060 responses analysed, from a total of 20 participants. Conditional r^2^: 0.340; Marginal r^2^: 0.163.

**Table S5**. ***LMM - Response criteria*.** Reference levels were set to red focus and blue context.

Model equation: C ~ 1 + attentional_focus + experiment + (1|participant)

| **Fixed Effects** | **Estimate ± SE** | **t value** | **Df** | **p value** |
| --- | --- | --- | --- | --- |
| Intercept | 0.397 ± 0.054 | 7.372 | 20 | <.001*** |
| Green vs. red focus | -0.002 ± 0.036 | 0.128 | 340 | 0.898 |
| Divided vs. red focus | 0.182 ± 0.036 | 4.991 | 340 | <.001*** |
| EEG vs. behavoural experiment | 0.072 ± 0.030 | 2.405 | 340 | 0.0167 |

Random effects: The maximal model that could be adequately fitted to the dataset included random by-participant intercepts, which accounted for 0.054 ± 0.231 in variance. Residual variance 0.08 (ICC = 0.40). There were 360 observations analysed, from a total of 20 participants. Conditional r^2^: 0.438; Marginal r^2^: 0.062.

**Note**: The three-way interaction did not contribute significantly to the model (χ^2^(4) = 0.804, p = 0.938), so it was removed; the same was the case for attentional focus by context (χ^2^(4) = 2.250, p = 0.690) context by experiment (χ^2^(2) = 0.230, p = 0.892) and attentional focus by experiment (χ^2^(2) = 1.227, p = 0.54). Finally, context could also be removed from the model (χ^2^(2) = 1.698, p = 0.428).

**Table S6**. ***LMM -* attentional effects on SSVEP amplitudes.** Reference levels were set to red focus and blue context.

Model equation: attended minus unattended target amplitude ~ 1 + attentional_focus + target_colour + colour_context +target_colour:colour_context + (1|participant)

|  | Attentional modulation (A-U) | | | |
| --- | --- | --- | --- | --- |
| *Predictors* | *Estimate* | *95% CI* | *t* | *p* |
| (Intercept) | 0.338 | 0.28 – 0.40 | 10.752 | <.001 |
| Attentional Focus (divided vs. single) | -0.169 | -0.23 - -0.11 | -5.801 | <.001 |
| Target Colour (green vs. red) | -0.109 | -0.17 - -0.05 | -3.757 | <.001 |
| Context (lime vs. blue) | 0.044 | -0.03 – 0.11 | 1.226 | 0.221 |
| Context (orange vs. blue) | -0.039 | -0.11 – 0.03 | -1.095 | 0.274 |
| Target Colour x Lime vs. blue context difference | -0.105 | -0.24 – 0.04 | -1.471 | 0.142 |
| Target Colour x Orange vs. blue context difference | 0.134 | -0.01 - 0.27 | 1.880 | 0.061 |
| *Random effects* |  | | | |
| Residual variance, σ^2^ = 0.051 |  |  |  |  |
| Participant variance 0.0156 (0.125 SD) |  |  |  |  |
| ICC 0.24 |  |  |  |  |
| N participant 20, 240 Observations in total |  |  |  |  |
| Marginal R^2^ / Conditional R^2^ 0.171 / 0.366 |  |  |  |  |

**Note**: The three-way interaction did not contribute significantly to the model (χ^2^(2) = 0.565, p = 0.754), so it was removed; the same was the case for attentional focus by context (χ^2^(2) = 0.581, p = 0.748) and attentional focus by colour (χ^2^(1) = 0.396, p = 0.529).

**Table S6.1** ***LMM -* attentional effects on SSVEP amplitudes – separate analysis for red.** Reference level set to blue context.

Model equation: attended minus unattended red amplitude ~ 1 + attentional_focus + colour_context + (1|participant)

|  | Attentional modulation (A-U) | | | |
| --- | --- | --- | --- | --- |
| *Predictors* | *Estimate* | *95% CI* | *t* | *p* |
| (Intercept) | 0.393 | 0.31 – 0.48 | 9.377 | <.001 |
| Attentional Focus (divided vs. single) | -0.187 | -0.26 - -0.11 | -5.135 | <.001 |
| Context (lime vs. blue) | 0.096 | 0.01 – 0.18 | 2.154 | 0.033 |
| Context (orange vs. blue) | -0.106 | -0.19 – -0.02 | -2.375 | 0.019 |
| *Random effects* |  | | | |
| Residual variance, σ^2^ = 0.040 |  |  |  |  |
| Participant variance 0.028 |  |  |  |  |
| ICC 0.42 |  |  |  |  |
| N participant 20, 120 Observations in total |  |  |  |  |
| Marginal R^2^ / Conditional R^2^ 0.187 / 0.526 |  |  |  |  |

**Note**: The two-way interaction did not contribute significantly to the model (χ^2^(2) = 1.257, p = 0.534), so it was removed.

**Table S6.2** ***LMM -* attentional effects on SSVEP amplitudes – separate analysis for green.** Reference level set to single focus.

Model equation: attended minus unattended green amplitude ~ 1 + attentional_focus + (1|participant)

|  | Attentional modulation (A-U) | | | |
| --- | --- | --- | --- | --- |
| *Predictors* | *Estimate* | *95% CI* | *t* | *p* |
| (Intercept) | 0.284 | 0.21 – 0.36 | 7.229 | <.001 |
| Attentional Focus (divided vs. single) | -0.150 | -0.22 - -0.08 | -4.103 | <.001 |
| *Random effects* |  | | | |
| Residual variance, σ^2^ = 0.040 |  |  |  |  |
| Participant variance 0.0241 |  |  |  |  |
| ICC 0.37 |  |  |  |  |
| N participant 20, 120 Observations in total |  |  |  |  |
| Marginal R^2^ / Conditional R^2^ 0.081 / 0.425 |  |  |  |  |

**Note**: The two-way interaction did not contribute significantly to the model (χ^2^(2) = 0.206, p = 0.902) and neither did context (χ^2^(2) = 0.731, p = 0.694), so they were removed.

**Table S7**. ***LMM -* SSVEP amplitudes elicited by yellow.** Reference levels were set to red focus and blue context.

Model equation: amplitude ~ 1 + attentional_focus + colour_context + (1|participant)

|  | Amplitude | | | |
| --- | --- | --- | --- | --- |
| *Predictors* | *Estimate* | *95% CI* | *t* | *p* |
| (Intercept) | 0.831 | 0.704 – 0.964 | -2.904 | <.001 |
| Attentional Focus: green vs. red | -0.083 | -0.115 - -0.052 | -4.361 | <.001 |
| Attentional Focus: divided vs. red | 0.0003 | -0.035 - 0.036 | -0.423 | 0.987 |
| Context (lime vs. blue) | 0.002 | -0.037 – 0.040 | -0.293 | 0.928 |
| Context (orange vs. blue) | -0.057 | -0.102– -0.013 | -2.927 | 0.003 |
| *Random effects* |  | | | |
| Residual variance, σ^2^ = 0.0228 |  |  |  |  |
| Participant variance 0.204 |  |  |  |  |
| N participant 20, 180 Observations in total |  |  |  |  |
| Marginal R^2^ / Conditional R^2^ 0.019 / 0.901 |  |  |  |  |

**Note**: The three-way interaction did not contribute significantly to the model (χ^2^(2) = 0.565, p = 0.754), so it was removed; the same was the case for attentional focus by context (χ^2^(2) = 0.581, p = 0.748) and attentional focus by colour (χ^2^(1) = 0.396, p = 0.529).

This model violated the assumption of normally distributed residuals, suffering from excessive outliers. To facilitate interpretation, we report estimates and confidence intervals from a bootstrapped model (with 1000 samples), obtained using the *lmeresampler* R package. We report p values and other properties of the model (e.g., residual variance and r squared) from the model fitted to log transformed amplitudes, which is compliant with all the assumptions and exhibits the same statistically significant effects.

**Table S8** ***LMM -* SSVEP amplitudes elicited by blue.**

Model equation: amplitude ~ 1 + (1|participant)

|  | Amplitude | | | |
| --- | --- | --- | --- | --- |
| *Predictors* | *Estimate* | *95% CI* | *t* | *p* |
| (Intercept) | 1.075 | 0.912 – 1.237 | 13.59 | <.001 |
| *Random effects* |  | | | |
| Residual variance, σ^2^ = 0.011 |  |  |  |  |
| Participant variance 0.121 |  |  |  |  |
| ICC 0.92 |  |  |  |  |
| N participant 20, 60 Observations in total |  |  |  |  |
| Marginal R^2^ / Conditional R^2^ 0 / 0.919 |  |  |  |  |

**Note**: Attentional focus did not contribute significantly to the model (χ^2^(2) = 2.712, p = 0.258).

**Table S9** ***LMM -* SSVEP amplitudes elicited by lime.**

Model equation: amplitude ~ 1 + (1|participant)

|  | Amplitude | | | |
| --- | --- | --- | --- | --- |
| *Predictors* | *Estimate* | *95% CI* | *t* | *p* |
| (Intercept) | 0.494 | 0.400 – 0.588 | 10.81 | <.001 |
| *Random effects* |  | | | |
| Residual variance, σ^2^ = 0.010 |  |  |  |  |
| Participant variance 0.038 |  |  |  |  |
| ICC 0.80 |  |  |  |  |
| N participant 20, 60 Observations in total |  |  |  |  |
| Marginal R^2^ / Conditional R^2^ 0 / 0.798 |  |  |  |  |

**Note**: Attentional focus did not contribute significantly to the model (χ^2^(2) = 4.793, p = 0.091).

**Table S10.** ***LMM -* SSVEP amplitudes elicited by orange.** Reference level was set to red focus.

Model equation: amplitude ~ 1 + attentional_focus + (1|participant)

|  | Amplitude | | | |
| --- | --- | --- | --- | --- |
| *Predictors* | *Estimate* | *95% CI* | *t* | *p* |
| (Intercept) | 0.921 | 0.72 – 1.12 | 9.347 | <.001 |
| Green vs. red | -0.097 | -0.164 - -0.030 | -2.894 | 0.006 |
| Divided vs. red | -0.097 | -0.164 - -0.030 | -2.895 | 0.006 |
| *Random effects* |  | | | |
| Residual variance, σ^2^ = 0.011 |  |  |  |  |
| Participant variance 0.190 |  |  |  |  |
| ICC 0.94 |  |  |  |  |
| N participant 20, 60 Observations in total |  |  |  |  |
| Marginal R^2^ / Conditional R^2^ 0.01 / 0.945 |  |  |  |  |
